# Regional Disparities in Domiciliary Dental-Care Utilization and Their Association with Pneumonia Mortality in Japan: An Ecological Study Using National Open Data

**DOI:** 10.31662/jmaj.2025-0561

**Published:** 2026-06-19

**Authors:** Kyunghee Lee, Hideaki Kawaguchi

**Affiliations:** 1 Graduate School of Medical and Dental Sciences, Institute of Science Tokyo, Tokyo, Japan; 2 Medical Research Center for Pre-Disease State (Mebyo) AI, Graduate School of Medicine, The University of Tokyo, Tokyo, Japan

**Keywords:** prevention, home care, pneumonia, older adults, socioeconomic status, healthcare

## Abstract

**Introduction:**

Pneumonia is the leading cause of death among older adults, and oral care contributes to pneumonia prevention. Disparities in access to dental care and socioeconomic factors may influence service use among community-dwelling older adults. Therefore, in this study, we examined the association between the regional utilization of domiciliary dental care (DDC) and pneumonia mortality, as well as the impact of area-level socioeconomic conditions.

**Methods:**

In this nationwide ecological cross-sectional study, we used publicly available data from 335 secondary medical areas across Japan in 2023. The primary outcome was the standardized mortality ratio (SMR) for pneumonia. The primary explanatory variable was the DDC utilization ratio, and the secondary analysis used per-capita claims (PCC). Area-level socioeconomic status (area SES) was defined using the area deprivation index. Healthcare resources were included as covariates, and a Bayesian spatial analysis was performed using a conditional autoregressive model.

**Results:**

Moran’s I for the SMR for pneumonia was 0.361 (p < 0.01). In the conditional autoregressive model, the DDC ratio was significantly associated with the SMR for pneumonia (relative risk [RR]: 1.04; 95% confidence interval [CI]: 1.002-1.08). However, area SES showed no significant association (RR: 1.05; 95% CI: 0.998-1.10). The PCC was also significantly associated with the SMR for pneumonia (RR: 1.06; 95% CI: 1.01-1.11), whereas area SES showed no significant association (RR: 1.05; 95% CI: 0.998-1.10). The PCC were weakly and inversely correlated with area SES.

**Conclusions:**

Both the DDC ratio and PCC were positively associated with the SMR for pneumonia, suggesting that DDC is concentrated among frail older adults at high risk. Socioeconomically deprived areas tended to have fewer claims per patient, implying that regional disparities hinder preventive and continuous dental care. Strengthening preventive interventions and improving access to support tailored to local health needs and socioeconomic contexts are required.

## Introduction

Pneumonia is a leading cause of death among older adults. Oral care has been shown to significantly reduce pneumonia in this population, and improvements in oral health can contribute to improved overall health and can enhance quality of life. Frail, community-dwelling older adults face multiple risks including poor oral health, diminished oral function, and restricted access to dental services ^(1), (2), (3)^. Owing to these vulnerabilities, homebound older adults represent a population of particular relevance for pneumonia prevention, and supporting their oral care is critical within the evolving framework of home-based medical care.

Although dental care plays an important role in maintaining oral function and in preventing pneumonia in older adults, its utilization remains challenging. For example, older adults with higher care-need levels tend to be more likely to use domiciliary dental services ^(4)^, and individuals with lower activities of daily living are more likely to desire home dental visits than their independent counterparts ^(1)^. Nevertheless, the actual utilization rate of domiciliary dental care (DDC) among long-term care beneficiaries remains as low as 8.2% ^(4)^, suggesting that the current service provision does not adequately meet the dental care needs of homebound older adults ^(3)^.

Socioeconomic factors may contribute to the low utilization of DDC. A study using Japanese long-term care data reported higher use of domiciliary dental services in areas with higher household income levels ^(4)^. Additionally, multiple large-scale studies, including those involving older adults, have consistently demonstrated associations between economic disadvantage and reduced dental-care use. For instance, a national survey in Sweden found that socially disadvantaged individuals were seven to nine times more likely to refrain from necessary dental treatment despite poor oral health ^(5)^, and a study in 11 European countries demonstrated significantly lower dental attendance among low-income groups ^(6)^. Furthermore, research among National Health Insurance beneficiaries in Japan has shown that individuals with lower incomes have lower dental care utilization ^(7)^, while studies on older adults have indicated that indicators of economic well-being, such as wealth and pension levels, better explain inequalities in dental attendance than income alone ^(8)^. Collectively, these findings suggest that economic hardship suppresses dental care–seeking behaviors. Such socioeconomic constraints may reduce dental service use among homebound older adults, hindering the provision of the oral care necessary for pneumonia prevention; however, this relationship has not yet been fully elucidated. Clarifying the association between dental service utilization and socioeconomic factors among homebound older adults, as well as its impact on regional pneumonia mortality, is essential from both public health and policy perspectives.

In this study, we aimed to use publicly available government data to examine nationwide associations among area-level socioeconomic conditions, utilization of DDC, and pneumonia mortality across all secondary medical areas (SMAs) in Japan. Through this investigation, we seek to contribute to the development of oral health policies for homebound older adults and to improve home-based healthcare systems at the regional level.

## Materials and Methods

### Study design

This was an ecological cross-sectional study of SMAs (n = 335) across Japan in 2023. The year 2023 was selected because the Ministry of Health, Labour and Welfare (MHLW) began releasing patient counts by fee schedule categories through the National Database (NDB) Open Data ^(9)^, enabling a more detailed analysis of DDC. SMAs were chosen as the unit of analysis because they represent the smallest geographic unit available in the NDB Open Data, consist of multiple municipalities, and serve as regional units for planning inpatient care and coordinating hospital bed supply ^(10), (11), (12), (13)^.

### Data sources

Data on home medical care and DDC were obtained from the 10th NDB Open Data (2023) published by the MHLW ^(9)^. The NDB Open Data provide aggregated counts of patients and claims by fee schedule categories. However, information on the specific content or quality of DDC was not included in the NDB Open Data. For DDC, we extracted patient counts for “C000: Dental Home-Visit Fee,” and for home medical care, we used “C001: Home-Visit Medical Care Fee (I).” Population and household composition data were obtained from the 2020 Population Census ^(14)^. Geographical information was obtained from the National Land Numerical Information download site ^(15)^. Pneumonia-related deaths were obtained from the 2023 Vital Statistics ^(16)^. The numbers of hospitals, clinics, physicians, and dentists were obtained from the 2023 Survey of Medical Facilities (static/dynamic) ^(17)^ and the 2022 Statistics of Physicians, Dentists, and Pharmacists ^(18)^. Some variables were available only at the municipal level; therefore, these data were aggregated at the SMA level. Municipal–SMA correspondences were confirmed using tables from the 2020 and 2023 Patient Surveys ^(19)^, ensuring consistency across years, and municipal-level data were summed to obtain SMA-level values.

### Primary outcome

The primary outcome was the standardized mortality ratio (SMR) for pneumonia. The SMR for pneumonia was calculated as the ratio of the observed number of pneumonia deaths in each SMA in 2023 to the expected number of pneumonia deaths estimated from the national age- and sex-specific pneumonia mortality rates and each SMA’s population structure.

### Primary explanatory variables

To capture the regional utilization of DDC among homebound older adults, we created a DDC utilization ratio (DDC ratio). This ratio was defined as the number of patients billed for “C000: Dental Home-Visit Fee” divided by the number of patients billed for “C001: Home-Visit Medical Care Fee.” This indicator reflects the relative extent to which dental care is provided within the overall home medical care system, and serves as a proxy for the level of dental service use among older adults requiring home-based care. Although the numerator and denominator of the DDC ratio do not represent identical populations, both C000 and C001 primarily capture homebound older adults requiring continuous medical care. Because C000 and C001 are not linked at the individual level, this ratio does not represent a true utilization rate within a single defined patient population. Therefore, we considered the DDC ratio as a reasonable proxy for the relative extent to which dental care is incorporated into the home medical care system in each region.

To assess service intensity, we defined the per-capita claims (PCC) as the number of claims for C000 divided by the corresponding number of patients. PCC reflects the frequency with which domiciliary dental services are provided per patient. Although it does not distinguish whether visits were preventive, therapeutic, or emergency, we considered it to serve as a valid indicator of service intensity and continuity.

### Covariates

Area-level socioeconomic status (area SES) was defined as the cumulative population proportion based on the areal deprivation index (ADI), with higher ADI values indicating greater deprivation ^(20), (21)^. An area’s SES ranged from 0 (least deprived) to 1 (most deprived) ^(22)^. The ADI was calculated from Population Census data [14] using the following formula ^(20), (21), (22)^:

*ADIi = k×(2.99×proportion of older couple householdsi + 7.57×proportion of older single householdsi + 17.4×proportion of single mother householdsi + 2.22×proportion of rental housesi + 4.03×proportion of sales and service workersi + 6.05×proportion of agricultural workersi + 5.38×proportion of blue collar workersi + 18.3×unemployment ratei)*

*where i is an area index (the area here being municipalities), and k is a positive constant and can be any positive number in determining the percentile rank of a municipality.*

To account for healthcare resource distribution, we included the number of hospitals, clinics, physicians, and dentists in each SMA, converted to values per 100,000 population ^(13), (23), (24)^.

### Statistical analysis

Descriptive statistics were calculated for SMA levels. Area SES was divided into quartiles (Q1-Q4), with higher quartiles indicating greater deprivation ^(22)^. The regional distributions of the DDC ratio, PCC, and area SES were visualized using QGIS (version 3.99-Master) ^(25)^. Pearson’s correlation coefficients were used to assess crude associations between area SES and the two dental care indicators.

### Main analysis

The main analysis examined the association between the utilization ratio and the SMR for pneumonia. A Poisson regression model was fitted with the observed number of pneumonia-related deaths as the dependent variable and the log of expected deaths as the offset. A negative binomial model was used to detect overdispersion. The covariates included area SES and healthcare resource indicators. Variance inflation factors (VIFs) were calculated in non-spatial models, with a VIF of more than 2.5 used as the exclusion criterion for multicollinearity. Moran’s I was computed for the SMR for pneumonia to test spatial autocorrelation; if significant, a Bayesian hierarchical spatial model using a conditional autoregressive structure (CAR model) was applied ^(13), (23), (26)^. Bayesian estimation was conducted using Markov Chain Monte Carlo (MCMC) with 50,000 burn-in iterations, 150,000 total iterations, and a single chain with a thinning of 1, following the recommendations that thinning is generally unnecessary for sampling efficiency ^(27)^. Convergence was evaluated using the Geweke diagnostic tool (|z| < 1.96) ^(28)^. Results are expressed as relative risks (RRs) with 95% confidence intervals (CIs). Model fit was assessed using the widely applicable information criterion (WAIC) ^(29)^.

### Secondary analysis

In the secondary analysis, the primary explanatory variable was replaced by the PCC, whereas all other model specifications, including covariates, model selection procedures, MCMC parameters, and convergence diagnostics, remained the same. This allowed us to assess the association between the frequency of domiciliary dental service provision and the SMR for pneumonia.

All analyses were performed using R software (version 4.4.3) ^(30)^. Maps were generated using QGIS (version 3.99-Master).

### Ethical considerations

This study used only publicly available, anonymized, and aggregated data with no personally identifiable information. Therefore, the requirements for approval by an ethics committee and for obtaining informed consent were waived.

## Results

### Descriptive statistics

Table 1 presents the descriptive statistics for the variables across 335 SMAs. The median SMR for pneumonia was 1.02 (0.85–1.26), indicating substantial variation among regions. The median DDC ratio (dental home-visit patients/home medical care patients) was 0.72 (0.48–1.04). The mean utilization ratio was lowest in Q1 (least deprived) areas (0.71 ± 0.32) and highest in Q4 (most deprived) areas (0.88 ± 0.65).

**Table 1.** Characteristics of 335 Secondary Medical Service Areas in Japan.

		Total population	SMR of pneumonia	Area SES	DDC ratio	PCC	Number of hospitals¶	Number of medical clinics¶	Number of physicians¶	Number of dentists¶
All (n = 335)	Mean (SD)	1,566,322.59 (2,738,703.06)	1.06 (0.32)	0.7 (0.26)	0.8 (0.49)	5.72 (2.68)	2.1 (1.03)	20.35 (4.45)	59.42 (21.5)	17.72 (4.88)
	Median (IQR)	856,964 (385,477-1,813,930)	1.02 (0.85-1.26)	0.77 (0.5-0.93)	0.72 (0.48-1.04)	5.49 (3.65-7)	1.86 (1.34-2.62)	19.79 (17.62-22.7)	53.84 (44.93-67.46)	16.99 (14.67-19.41)
Q1 (n = 84)	Mean (SD)	3,102,332.31 (4,629,054.18)	0.96 (0.27)	0.31 (0.13)	0.71 (0.32)	6.42 (2.18)	1.36 (0.6)	19.9 (4.5)	64.42 (23.72)	19.37 (5.72)
	Median (IQR)	2,010,492 (1,271,471-3,322,835)	0.95 (0.77-1.13)	0.33 (0.24-0.42)	0.68 (0.51-0.86)	6.72 (4.73-8.33)	1.24 (0.96-1.59)	19.62 (16.83-21.91)	56.77 (47.21-79.91)	17.82 (15.33-21.14)
Q2 (n = 84)	Mean (SD)	1,695,026.14 (1,647,939.64)	1.02 (0.3)	0.66 (0.08)	0.84 (0.48)	6.1 (2.54)	1.78 (0.6)	20.28 (3.6)	64.05 (22.66)	18.4 (4.85)
	Median (IQR)	1,363,468 (757,314-1,867,499)	0.95 (0.86-1.16)	0.67 (0.59-0.73)	0.76 (0.52-1.06)	5.98 (3.99-7.18)	1.73 (1.38-2.05)	19.7 (17.78-22.33)	59.16 (47.18-75.45)	17.69 (15.32-19.78)
Q3 (n = 83)	Mean (SD)	1,043,632.31 (1,399,454.31)	1.13 (0.33)	0.86 (0.05)	0.78 (0.43)	5.3 (2.75)	2.28 (0.94)	20.82 (4.6)	57.89 (21.21)	17.29 (4.33)
	Median (IQR)	540,576 (324,432-1,102,376)	1.14 (0.91-1.35)	0.87 (0.82-0.9)	0.76 (0.46--0.95)	4.78 (3.32-6.46)	2.12 (1.63-2.63)	19.86 (18.39-22.92)	52.5 (43.69-63.68)	16.99 (14.46-18.83)
Q4 (n = 84)	Mean (SD)	418,077.1 (303,175.05)	1.14 (0.34)	0.97 (0.02)	0.88 (0.65)	5.06 (3.02)	2.99 (1.08)	20.41 (5.04)	51.29 (14.96)	15.8 (3.75)
	Median (IQR)	300,964 (210594.5-530093)	1.09 (0.89-1.29)	0.97 (0.95-0.99)	0.72 (0.43-1.24)	4.57 (3.04-6.21)	2.9 (2.08-3.58)	20.56 (16.67-23.54)	50 (40.12-58.4)	15.21 (13.03-17.84)

Area SES: area-level socioeconomic status; DDC ratio: domiciliary dental care utilization ratio; IQR: interquartile range; PCC: per-capita claims; SD: standard deviation; SMR: standardized mortality ratio. ¶: per 100,000 population.

In contrast, the mean PCC value was highest in Q1 (6.42 ± 2.18) areas and lowest in Q4 (5.06 ± 3.02) areas.

### QGIS

**Figure 1.**
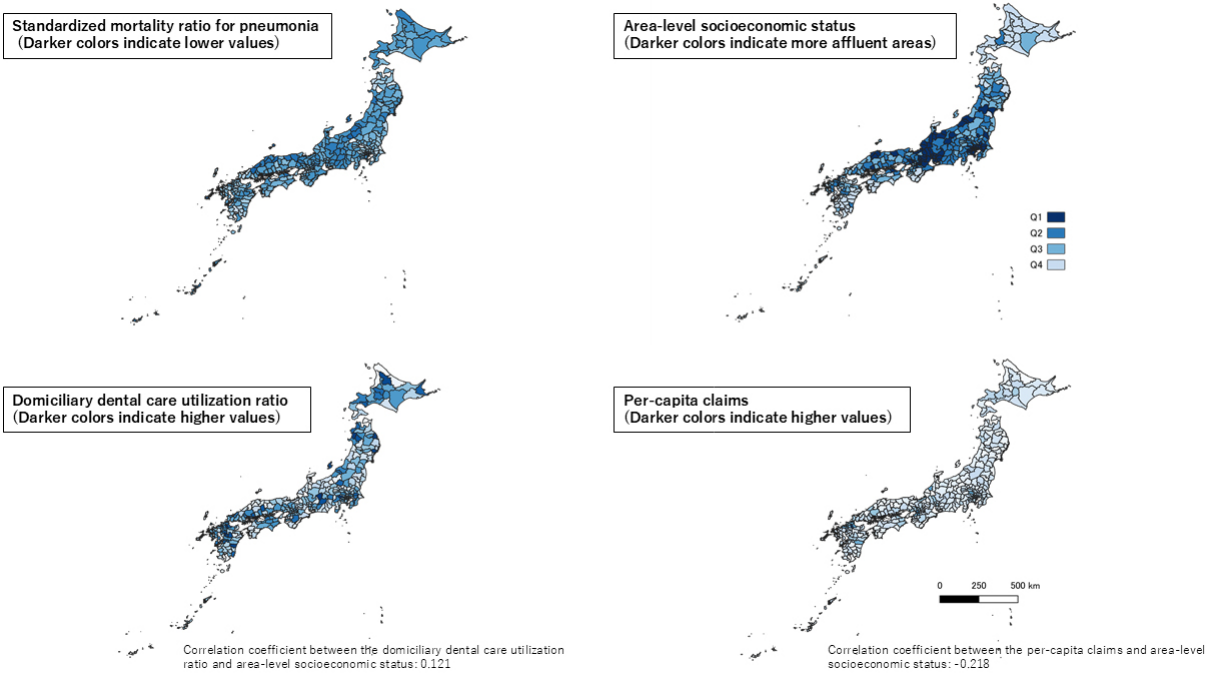
Geographical distributions of the standardized mortality ratio of pneumonia, area-level socioeconomic status, domiciliary dental care utilization ratio, and per-capita claims across secondary medical areas in Japan. This composite figure consists of four maps illustrating regional variations in key variables used in the analysis. The SMR map shows the ratio of observed to expected pneumonia deaths in 2023, calculated using national age- and sex-specific pneumonia mortality rates. The area SES map represents socioeconomic deprivation based on the areal deprivation index. The DDC ratio map shows the proportion of patients billed for dental home-visit fees relative to those billed for home medical care fees, and the PCC map displays the number of claims for dental home-visit fees per patient. Correlations between area SES and each dental-care indicator (DDC ratio and PCC) are also presented within the figure to visually demonstrate their relationships. DDC: domiciliary dental care; PCC: per-capita claims; SES: socioeconomic status; SMR: standardized mortality ratio.

Figure 1 shows the geographic distributions of SMR for pneumonia, area SES, DDC ratio, and PCC. The DDC ratio exhibited notable regional variations, tending to be higher in urban areas. The area SES values were lower (less deprivation) in urban regions and higher (greater deprivation) in rural areas, particularly in Hokkaido and Kyushu. The correlation coefficient between the DDC ratio and the area SES was 0.121, indicating no clear association. The PCC tended to be higher in urban areas and lower in rural regions. The correlation coefficient between the PCC and the area SES was −0.218, indicating that areas with higher area SES values (more deprived) tended to have lower PCC.

The SMR for pneumonia demonstrated a significant spatial autocorrelation across the SMAs, with a Moran’s I value of 0.361 (p < 0.01).

### Main analysis

Because overdispersion was observed in the Poisson regression model (dispersion ratio = 24.98), and the SMR for pneumonia exhibited spatial autocorrelation, we selected the CAR model. Model comparisons indicated that the CAR model provided the best fit (WAIC: CAR model = 2,853, negative binomial = 3,510, Poisson = 12,983). Table 2 summarizes the estimated effects and WAIC values of the three models.

**Table 2.** Results of the Comparison among CAR, Negative Binomial, and Poisson Regression Models for the Standardized Mortality Ratio for Pneumonia Using DCC Ratio.

	CAR model	Geweke diagnostic	Negative binomial model	Poisson regression model
	RR (95% CI)		RR (95% CI), p Value	RR (95% CI), p Value
Intercept	1.02 (1.01-1.03)	1.41	1.06 (1.03-1.09), <0.001**	1.07 (1.06-1.08), <0.001**
DDC ratio	1.04 (1.002-1.08)	0.27	1.06 (1.02-1.09), 0.002**	1.00 (0.99-1.01), 0.444
Area SES	1.04 (0.98-1.10)	−0.76	1.05 (1.00-1.09), 0.035*	1.12 (1.11-1.14), <0.001**
Number of hospitals¶	1.09 (1.04-1.15)	1.44	1.05 (1.01-1.09), 0.015*	1.07 (1.06-1.08), <0.001**
Number of medical clinics¶	1.02 (0.97-1.07)	0.84	0.97 (0.94-1.01), 0.182	1.06 (1.05-1.07), <0.001**
Number of physicians¶	0.96 (0.92-1.01)	−0.15	0.97 (0.93-1.01), 0.129	0.94 (0.93-0.94), <0.001**
Number of dentists¶	1.04 (0.99-1.09)	−0.58	1.04 (0.99-1.08), 0.090	1.06 (1.05-1.07), <0.001**
WAIC	2,853		3,510	12,983

Area SES: area-level socioeconomic status; CAR model: a conditional autoregressive model, which is a hierarchical Bayesian spatial model; CI: confidence interval; DDC ratio: domiciliary dental care utilization ratio; RR: relative risk; WAIC: widely applicable information criterion. *p < 0.05, **p < 0.01. ¶: per 100,000 population.

In the CAR model, the RR associated with the DDC ratio was 1.04 (95% CI: 1.002-1.08), demonstrating a significant positive association with the SMR for pneumonia. Area SES was not significantly associated with SMR for pneumonia (RR: 1.04; 95% CI: 0.98-1.10).

The Geweke statistics for all MCMC parameters were within |1.96|, indicating convergence. Multicollinearity, assessed using the negative binomial model, showed VIF values between 1.13 and 1.99.

### Secondary analysis

In the secondary analysis, the primary explanatory variable was replaced with PCC. As in the main analysis, overdispersion was observed in the Poisson model (dispersion ratio = 24.40), and the SMR for pneumonia showed spatial autocorrelation; therefore, the CAR model was adopted again. The model comparison results showed that the CAR model provided the best fit (WAIC: CAR model = 2,854, negative binomial = 3,503, Poisson = 13,189). Table 3 presents the estimates and WAIC values for the three models.

**Table 3.** Results of the Comparison among Bayesian CAR (Leroux), Negative Binomial, and Poisson Regression Models for the Standardized Mortality Ratio for Pneumonia Using PCC.

	CAR model	Geweke diagnostic	Negative binomial model	Poisson regression model
	RR (95% CI)		RR (95% CI), p Value	RR (95% CI), p Value
Intercept	1.02 (1.01-1.03)	0.93	1.086 (1.03-1.09), <0.001**	1.06 (1.05-1.07), 0.001**
PCC	1.06 (1.01-1.11)	0.62	1.08 (1.04-1.12), <0.001**	1.05 (1.04-1.06), 0.001**
Area SES	1.05 (0.998-1.10)	1.42	1.06 (1.02-1.10), 0.007**	1.12 (1.11-1.13), 0.001**
Number of hospitals¶	1.09 (1.05-1.15)	1.42	1.06 (1.02-1.10), 0.004**	1.08 (1.07-1.10), 0.001**
Number of medical clinics¶	1.01 (0.97-1.06)	−0.72	0.97 (0.93-1.01), 0.115	1.06 (1.05-1.07), 0.001**
Number of physicians¶	0.96 (0.92-1.01)	1.05	0.97 (0.93-1.01), 0.124	0.93 (0.92-0.94), 0.001**
Number of dentists¶	1.03 (0.98-1.08)	−0.17	1.02 (0.97-1.06), 0.449	1.04 (1.03-1.05), 0.001**
WAIC	2,854		3,503	13,189

Area SES: area-level socioeconomic status; CAR model: a conditional autoregressive model, which is a hierarchical Bayesian spatial model; CI: confidence interval; PCC: per-capita claims; RR: relative risk; WAIC: widely applicable information criterion. *p < 0.05, **p < 0.01. ¶: per 100,000 population.

PCC were significantly associated with the SMR for pneumonia (RR: 1.06; 95% CI: 1.01-1.11). Area SES was not significantly associated with the SMR for pneumonia (RR: 1.05; 95% CI: 0.998-1.10).

All Geweke statistics met the convergence criterion (|z| < 1.96). Multicollinearity, evaluated using a negative binomial model, yielded VIF values between 1.35 and 2.13.

## Discussion

In this nationwide ecological study of Japan’s SMAs, we examined the association between the regional utilization of DDC and the SMR for pneumonia using publicly available data and spatial statistical modeling. Both the DDC ratio and PCC were significantly and positively associated with the SMR for pneumonia in the CAR model, even after adjusting for geographic factors. These findings suggest that the concentration of medical services in high-need areas persists, despite accounting for spatial dependency. The PCC measure also showed a weak negative correlation with area SES, raising the possibility that socioeconomic disparities influence the access to and availability of domiciliary dental services within home care.

In this study, we found that regions with higher utilization ratios and higher per-capita provision of DDC tended to exhibit a higher SMR for pneumonia. This seemingly paradoxical positive association should not be interpreted as a harmful effect of dental interventions; rather, it may reflect the concentration of services among older adults who are at an elevated risk for pneumonia. The phenomenon in which greater healthcare resource input does not correspond to lower mortality has been noted historically ^(31)^ and is attributed to the clustering of healthcare delivery in high-need areas ^(32), (33)^. Though previous research has suggested similar supply–demand dynamics, few studies have applied spatial statistical analysis to explicitly account for geographic dependency. By adopting a CAR spatial model, our study demonstrated that these associations persisted, even after adjusting for spatial autocorrelation, suggesting that DDC is delivered primarily to older adults with substantial care needs ^(1), (4)^; however, this finding does not indicate the preventive effectiveness of DDC against pneumonia. Taken together, these findings imply that the expansion of domiciliary dental services should not focus solely on increasing service volume; instead, service allocation should be optimized according to regional care needs. This perspective is consistent with prior research emphasizing that the relationship between healthcare service volume and health outcomes must be evaluated with careful consideration of broader social determinants ^(32), (34)^.

Area SES showed a positive association with pneumonia mortality; however, this association did not reach statistical significance. Previous studies have shown that socioeconomic factors are independent risk determinants of respiratory disease mortality, and that income disparities and social deprivation are associated with increased respiratory disease incidence and mortality ^(21), (35), (36)^. Prior studies have focused on specific regions or adult populations ^(2), (5), (7), (21), (35), (36)^, and few have examined these associations on a nationwide scale using regional units that include homebound older adults. By conducting analyses across all Japanese SMAs, this study provides nationwide evidence suggesting that socioeconomic disadvantages may be related to pneumonia mortality at the regional level. However, because this association did not reach statistical significance even in the final CAR model, it should be interpreted with caution, and future studies using individual-level socioeconomic indicators and longitudinal designs are needed to evaluate these relationships in greater detail.

The observed trend of lower PCC in more-deprived regions may reflect a lack of regular dental care habits. Previous studies among older adults indicated that those with a higher SES were more likely to undergo preventive dental checkups ^(8)^. Previous studies on adults in Japan have shown that low-income regions have fewer preventive treatments such as scaling ^(37), (38)^ and more invasive treatments such as extractions ^(38)^. Our findings echo these earlier results, suggesting that preventive and continuous dental care may be difficult to maintain for older adults in socioeconomically disadvantaged regions. As our PCC measure was based on claims for dental home visit fees, it could not distinguish whether visits were preventive, therapeutic, or emergency.

Previous studies have shown that exemptions from out-of-pocket payments can increase the use of DDC ^(4)^. Therefore, it was expected that economic support systems, such as public assistance, would increase service frequency in more deprived regions; however, this association was not observed in our study. One possible explanation for this is that the overall utilization rate of DDC services remains low nationwide, resulting in limited regional variation. National data indicate that regional disparities in dental home-visit services are substantially larger than those in outpatient dental care, with differences reaching up to approximately 19-fold across regions ^(38)^. The national utilization rate of DDC among long-term care beneficiaries remains only 8.2% ^(4)^. Taken together, these findings suggest that structural factors related to regional maldistribution may not have been fully captured by this model and may have contributed to residual confounding in the observed associations. These findings suggest that regional maldistribution may continue to constrain access to domiciliary dental services.

Preventive dental care is more common among men with higher income levels ^(37)^. Furthermore, based on analyses of the Comprehensive Survey of Living Conditions ^(39)^ conducted by Japan’s MHLW, unmarried men are significantly less likely to receive dental treatment than their married counterparts, whereas this pattern is not observed among women ^(40)^. These findings indicate that social factors, such as sex, marital status, and family structure, may influence dental care-seeking behaviors.

This study had a few limitations. First, as an ecological study, the results reflect area-level associations and do not directly infer individual-level causal relationships. Accordingly, the findings should be interpreted at the SMA level and may be subject to ecological fallacy. Second, because the analysis was cross-sectional and based on a single year (2023), the temporal sequence between DDC utilization and pneumonia mortality cannot be established; therefore, causal inference is not possible and reverse causality cannot be excluded. Third, because the NDB Open Data provide only aggregated counts, we could not assess the specific content or quality of DDC provided. In addition, as the DDC ratio was constructed from two different fee categories that do not represent identical patient populations, it should be interpreted as an area-level proxy indicator rather than as a true utilization rate within a single defined population. Fourth, some variables were available only at the municipal level and had to be aggregated to the SMA level, which may have introduced measurement error. Finally, pneumonia-related mortality is influenced by multiple factors, including lifestyle-related factors such as smoking, immune function, vaccination status, and nutritional status, which were not available in the public aggregated data; therefore, residual confounding is possible. Future studies should incorporate individual-level public datasets, examine specific dental interventions, and conduct intervention-based studies to clarify the mechanisms linking DDC to health outcomes.

In this nationwide study of SMAs in Japan, we used publicly available data to comprehensively examine the regional utilization patterns of DDC and their association with mortality from pneumonia. Both the proportion of DDC within home medical services and the per-capita frequency of dental home-visit claims were significantly associated with the SMR for pneumonia, suggesting that domiciliary dental services may be concentrated among frail older adults at high risk for pneumonia. Additionally, the lower per-capita service frequency in more-deprived regions indicates that preventive and continuous dental care may be less accessible in socioeconomically disadvantaged areas. Strengthening preventive interventions and access to support that reflect regional health needs and socioeconomic contexts, and establishing evaluation methods to assess their effectiveness, may be crucial for improving oral health care within home medical systems.

## Article Information

### Acknowledgments

The authors would like to express their sincere gratitude to the faculty and administrative staff of The General Course of Medical Real World Data Utilization Human Resource Development Project, an extended educational program by The University of Tokyo, for their valuable guidance. We would also like to thank Editage (www.editage.com) for their writing support on the manuscript.

### Author Contributions

Conceptualization: all authors; methodology: all authors; data management & analysis: Kyunghee Lee; writing—original draft: Kyunghee Lee; writing—review & editing: all authors.

### Conflicts of Interest

None

### IRB Approval Code and Name of the Institution

This study used only publicly available, anonymized, and aggregated data with no personally identifiable information. Therefore, the requirements for approval by an ethics committee and for obtaining informed consent were waived.

### Data Availability Statement

All data used in this study are publicly available from the Ministry of Health, Labour and Welfare of Japan and other governmental sources. Individual-level data were not used.
